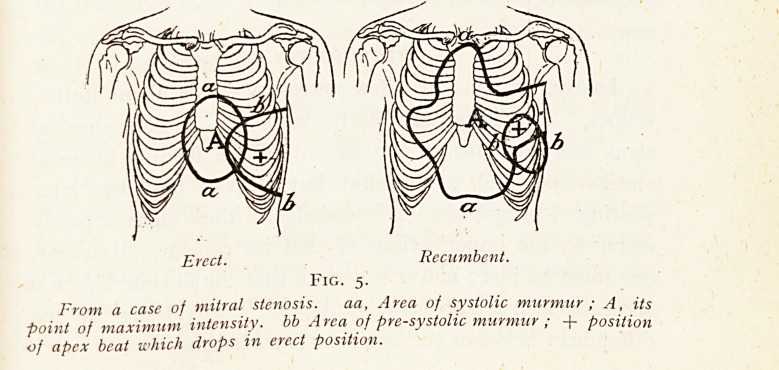# The Practical Importance of Posture in the Physical Examination of the Heart, with Some Remarks on Stethoscopes

**Published:** 1925

**Authors:** W. Gordon

**Affiliations:** Senior Physician to the Royal Devon and Exeter Hospital


### THE PRACTICAL IMPORTANCE OF POSTURE IN THE PHYSICAL EXAMINATION OF THE HEART, WITH SOME REMARKS ON STETHOSCOPES.

BY

W. GORDON, M.D., F.R.C.P.,

*Senior Physician to the Royal Devon and Exeter Hospital.*

---

MANY years of observation have made it abundantly clear that the effects of posture on the cardiac physical signs are matters of great importance, whether in the first diagnosis of a case or in the subsequent following of its fortunes. The old advice, sound though it was, to examine all hearts both in the erect and recumbent positions, had so little argument attached to it that practically no one regarded it. To-day there is so much good reason to back its repetition, that nothing but defective hearing can account for its neglect.

I propose briefly to summarise here the chief practical results arrived at since 1902. The dulness I am speaking of is sometimes called "the deep dulness." The so-called "superficial dulness," indicating the area uncovered by lung, has little practical importance. In "change of posture" I confine myself to the change from the erect to the recumbent and *vice versa*.

### THE SIZE OF THE HEART.

There are few things more necessary to determine in a case of heart disease than the actual size of the heart. I think no one will dispute this. Therefore it must be desirable to know how best to determine it. So I begin with the following :—

1. *The size of the heart, in the great majority of cases, can only be satisfactorily determined by percussion in the erect position.*

One sometimes sees it stated that the size of the heart is best determined by inspection and palpation. But in many cases the apex-beat is not palpable ; and although marked epigastric pulsation may indicate an enlarged right ventricle, it cannot tell us the extent of its enlargement. Again, though a general precordial heave (or, in children, a precordial bulging) points to a large heart, it does not define its limits. Finally, and especially, neither inspection nor palpation can give, in the great majority of cases, the slightest clue to the real position of the right border of the heart, nor can they distinguish between cardiac enlargement and displacement. Therefore by percussion only, of physical signs, can the size of the heart be satisfactorily determined in the great majority of patients. The sole alternative, X-ray orthodiagraphy, is at present only available for a small minority of cases. Even when pulmonary emphysema is present, it is surprising what approximate results percussion can give.

But in the recumbent posture matters are very different. In that position both borders of the heart tend to drop away from contact with the anterior chest-wall ; the thicker-walled left ventricle slightly, the thinner-walled right ventricle (especially when distended and heavy with blood) greatly. So that we arrive at the following conclusion :—

2. *In the recumbent posture, in the great majority of cases, percussion cannot satisfactorily determine the size of the heart and, on the right side, may gravely mislead us.*

A distended right ventricle may actually give no indication of its enlargement when percussed out in recumbency. Only in two sorts of case have I found the cardiac dulness identical in the erect and recumbent postures, viz. (1) where a heart is so large that it is wedged between the sternum and the spine, and (2) where a heart is pinned against the anterior chest-wall, either by adhesions in front or by pressure from behind.

Now to illustrate the foregoing.

Fig. 1 shows how closely an enlarged heart can be mapped out by percussion in the erect position. The thick straight lines are the X-ray shadows of copper wires fastened on the chest-wall over the pencilled limits of the percussion-dulness ;

FIG. 1.

*Marked and photographed both in the standing and recumbent positions. Only the erect heart outlines are given.*

the thin curved lines are the outlines of the cardiac shadow on the screen. The same figure shows the failure of percussion in the recumbent posture, especially on the right side.[1]

Fig. 2 shows the usual relations of the cardiac erect and recumbent dulness to each other in a normal person. The difference between the limits of dulness on the left is usually

[1] Exact percussion needs no exceptional powers. I have seen too many indifferent percussers learn to percuss well to have any doubt on that subject. Good percussion simply requires constant practice and close attention. Unless there is defective hearing or unhappily constructed fingers, it is within the reach of anyone.

about a quarter of an inch; the difference on the right is generally from half to three-quarters of an inch.

Fig. 3 shows the same relations in a case of dilated heart. Here the differences may be very great. In three cases

FIG. 2.

*Normal heart dulness; inner area A dulness in recumbency; B C outline of dulness in erect position. The latter is somewhat wider than usual.*

FIG. 3.

*From a case of hypertrophy and dilatation of the heart. A inner area, dulness in recumbency. B C the outline of dulness in the erect position. B C is not always higher than A.*

which I have instanced the differences in the total width of dulness were as follows (in inches):—

| | *Erect.* | *Recumbent.* |
|---|---|---|
| Case A .. .. .. .. | 9 | 5 |
| Case B .. .. .. .. | $8\frac{1}{4}$ | $4\frac{1}{2}$ |
| Case C .. .. .. .. | 8 | 5 |

Such total differences are nearly always due for the most part to the changes in the rightward limit of dulness. At the left of an enlarged heart there is rarely more than an inch of difference between the limits of the dulness in the two positions.

We are thus forced to the third conclusion, that:—

3. *A diseased heart must be always examined in the same posture, if possible in the erect posture.*

It is obvious that if the three patients above referred to had been examined, sometimes in the erect posture, sometimes in the recumbent, indiscriminately, the greatest confusion must have been caused in one's judgment of their progress.

### THE LEVEL OF THE HEART.

In some cases the heart drops abnormally in the chest when the erect position is assumed, so that the apex-beat, which in recumbency is found in the fifth space, is found in the sixth space when the patient stands ("cardioptosis"). I have seen such a case mistaken for dilatation. In order to avoid this error :—

4. *The position of the apex-beat should be observed in both postures.*

### MURMURS.

#### 1. *Organic.*

A little while ago the announcement that murmurs were of no importance, that so long as the cardiac muscle was sound the valves were of little account, and that our stethoscopes might be scrapped, was acclaimed with some enthusiasm. However, the hope proved delusive. The stethoscope has come back into its own, and we have witnessed the unobtrusive rediscovery of mitral stenosis and aortic regurgitation, with murmurs which matter very much. After all, cardiac sounds demand as much attention as other phenomena of disease. I would therefore emphasise the following :—

5. *Organic systolic murmurs are nearly always loudest in recumbency, and are sometimes only heard in that position.*

Mitral regurgitation is a typical example. When in doubt as to the existence of a mitral murmur, it is wise to always re-examine the patient recumbent. Fig. 4 shows the area of a mitral murmur which could be only heard in recumbency.

FIG. 4.

*Area of systolic mitral murmur after acute rheumatism in the recumbent position. The murmur was inaudible in the erect position.*

6. *The murmur of aortic regurgitation is practically unaffected by change of position.*

The occasional importance of this fact lies in the power which it gives us of distinguishing between a diastolic aortic murmur and a soft diastolic friction sound. The sound of friction, as is well known, sometimes vanishes on change of posture from erect to recumbent or *vice versa*. I have recorded a case in which it was possible, by means of this difference, to arrive at a correct diagnosis otherwise impossible.

*Erect.* *Recumbent.*

FIG. 5.

*From a case of mitral stenosis. aa, Area of systolic murmur; A, its point of maximum intensity. bb Area of pre-systolic murmur; + position of apex beat which drops in erect position.*

7. *The pre-systolic murmur of mitral stenosis, on the other hand, may be sometimes only audible in the erect position. It is the only cardiac murmur which has this peculiarity. But there are instances in which this murmur is better heard in recumbency.*

Fig. 5 illustrates a case in which posture modified the area of audibility (*i.e.* the loudness) of pre-systolic and systolic mitral murmurs in opposite directions.

### 2. *Hæmic Murmurs.*

8. *Hæmic murmurs are even more markedly affected by posture than "organic" murmurs, often becoming greatly louder in recumbency.*

So much is this the case that it has some value in diagnosis. A systolic murmur faintly heard in the erect posture, which becomes *extremely* loud in recumbency is generally "hæmic."

### 3. *"Respiratory" murmurs.*

9. *So-called "respiratory" murmurs commonly vanish on recumbency.*

This is a useful fact and, in conjunction with the other recognised characteristics of these murmurs, enables us to distinguish them from those which are "organic."

### STETHOSCOPES.

Lastly, let me add a few necessary words about stethoscopes. The important early work on cardiac physical signs was done before the advent of the flexible binaural stethoscope, and one cannot but suspect that the later slowing of progress in our knowledge of these signs is partly owing to the imperfections of that instrument. However, one must be just; and it is certain that the flexible binaural stethoscope has also its advantages. I would sum up the differences between the two instruments thus:—

(*a*) Some people can scarcely hear anything with a straight wooden stethoscope.

(*b*) On the other hand, some people hear very little through their binaural stethoscopes, because the ear-pieces do not fit their ears.

(*c*) The straight wooden stethoscope can detect a slight aortic regurgitant murmur which the flexible stethoscope cannot. This is not rare. Here I would recall the fact that sometimes an aortic regurgitant murmur may be inaudible through both instruments, yet be audible to the unaided ear laid direct on the chest. The French are wise in using such "immediate" auscultation much more than we do.

(*d*) The flexible stethoscope often makes a mitral systolic murmur more distinct than the wooden one does.

(*e*) For tracing the "conduction" of a murmur, for determining its point of maximum intensity on the chest, and for comparing its intensity from time to time, the flexible stethoscope, whose adaptation to one's ears varies with movement, is less reliable than the wooden one.

(*f*) (Although not a heart question, this needs mention in this comparison.) In a lung case the flexible stethoscope appears to have the power of penetrating more deeply into the lung, and so of picking up deeply-produced sounds which the wooden stethoscope may wholly miss. Therefore we come to this last result, viz. :—

10. *We should use both the straight wooden and the flexible binaural stethoscopes as a routine in examining our cases.*

In conclusion, let me urge the certainty that percussion and auscultation are as important to-day as they ever were, before the advent of additional methods of investigation, and that to the older use of them must be now added the knowledge of those important changes in their results which are produced by change of the patient's posture.

## Figures and Tables

**Fig. 1. f1:**
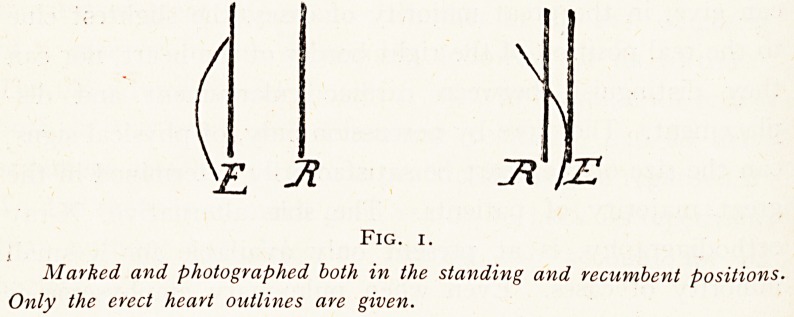


**Fig. 2. f2:**
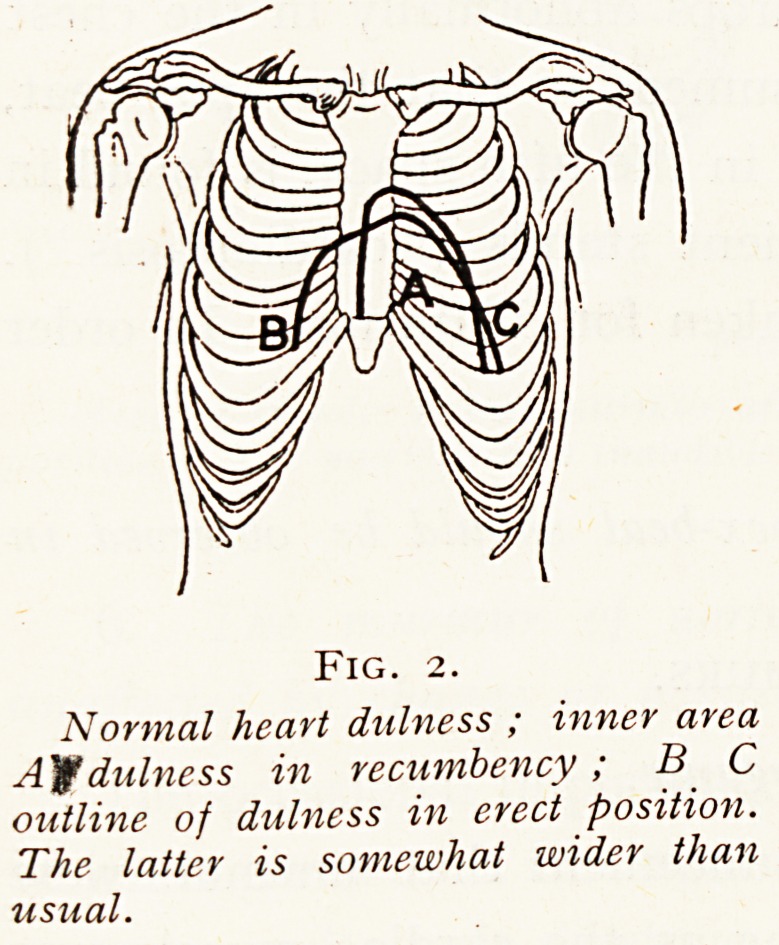


**Fig. 3. f3:**
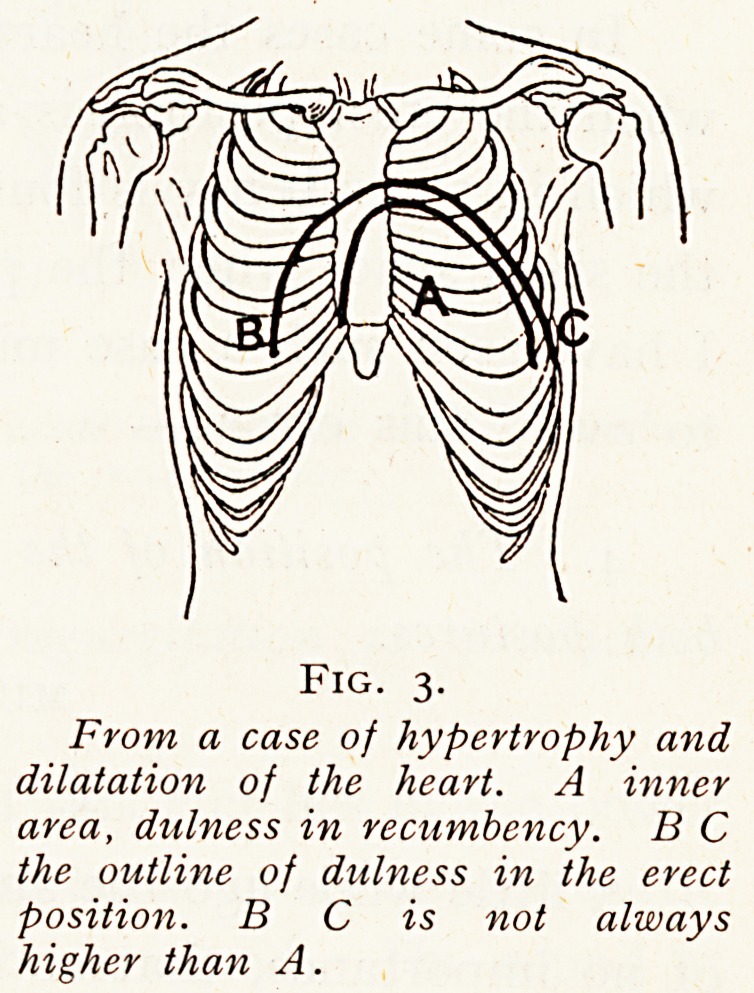


**Fig. 4. f4:**
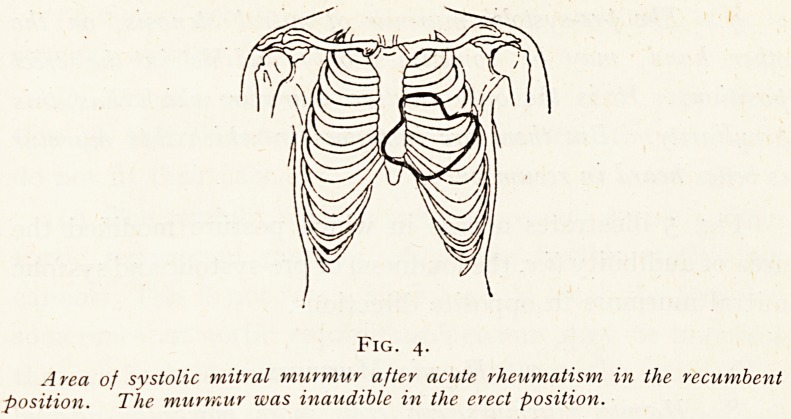


**Fig. 5. f5:**